# Open Access for *The Clinical Respiratory Journal*

**DOI:** 10.1111/crj.13477

**Published:** 2022-01-27

**Authors:** Paul Jones, Chen Wang

**Affiliations:** ^1^ St George's, Institute for Infection and Immunity University of London London UK; ^2^ Chinese Academy of Medical Sciences & Peking Union Medical College Beijing China; ^3^ National Center for Respiratory Medicine Beijing China; ^4^ Institute of Respiratory Medicine Chinese Academy of Medical Sciences Beijing China; ^5^ National Clinical Research Center for Respiratory Diseases Beijing China

*The Clinical Respiratory Journal* is becoming Open Access from its first issue of 2022. This means that all articles accepted for publication will be published under the terms of the Creative Commons Attribution License (CC BY). This permits use, distribution and reproduction of the article in any medium, provided the original work is properly cited. It means that all the content of CRJ articles will be free of charge to the user, but also means that all articles will be subject to an article publication charge (APC) to cover the cost of publishing.

Whilst authors may think this could be a barrier to getting their work published, it is in fact an exciting platform that will facilitate greater access to CRJ papers and a wider audience as a result. Experience has shown that when a journal becomes open access, it increases the visibility of the authors' work and the journal's impact. For authors who have difficulties paying the APC, there are policies to support them. APC waivers are provided to authors in Research4Life countries and eligible institutions that have signed a Transitional Deal with the publisher, Wiley.

Currently, a potential journal reader must depend on the title and abstract to make a judgement about whether they need to read the paper, but whilst the CRJ tries to ensure that the title and abstract are informative, they only provide a narrow window into the paper's contents. The reader will not see a very illustrative figure or read a thought‐provoking interpretation of the results. With Open Access, they can immediately see the full contents. Since September, we have already asked authors of original articles, review articles, brief reports and mini reviews to submit a graphical abstract during initial submission; this includes an image and brief introduction to the key information and findings of the paper. These can help users locate papers of interest when they browse our Table of Contents.

These developments provide an opportunity to think about the position of the CRJ as a clinically focussed general respiratory journal, when so many subspecialty journals are now available. We believe that it still has an important role to fulfil demands from the general pulmonologist and respiratory medical researcher. One only has to look at the major specialty journals and see that there may be only one paper from a specific sub‐specialty such as asthma and, in some months, none at all. Yet an asthma specialist still reads the journal, and the reason is simple—respiratory medicine is more than a collection of unrelated sub‐specialties. The very great majority of pulmonologists are not super‐specialists who are only interested in the latest cutting‐edge research.

CRJ's editorial philosophy is to publish papers that may have an impact on medical practice and clinical research now, or in the not‐too‐distant future. Occasionally, a basic science paper is published because it may provide a long‐term perspective view of new and important developments in the future, but CRJ is not usually the natural home for such papers. Journal editors have two main roles—as journalists to identify new and interesting developments and as scientists to ensure that they publish reliable peer‐reviewed research and evidence. Research papers are of two kinds, one reports brand new and innovative findings, but these are relative rare; the others are studies that test the reliability and generalisability of previously reported findings. That type of paper is extremely important; one only has to look at any meta‐analysis and see the degree of heterogeneity between studies. All findings need to be tested and replicated—something that is fundamental to the scientific process. At the same time, no one wants to read replicate ‘me too’ studies of the same topic. CRJ will, of course, publish papers that help confirm the reliability of previous findings, but at the same time, such papers also need to add their own original contribution that will advance insights into that area of medicine.

Editors and reviewers make important decisions, but they are human and like the reassurance of familiarity. It sometimes takes courage to publish a paper that seems ‘way out’ of current thinking, even if the methodology and science look sound. The CRJ is prepared to make that decision, if the methodology looks strong, knowing that the findings will need to be tested in further studies. Pilot studies may come in that category, but ‘pilot’ does not mean small and underpowered, it means that the study is designed to test the feasibility of a bigger definitive study. It should be set up to answer specific *a priori* questions about feasibility. CRJ is interested in proper pilot studies because they are looking to the future.

Over the last few years, the CRJ has made significant strides:

- Published its 1500th paper in 2021.
- Became online‐only and bi‐monthly publishing in 2016.
- Started publishing monthly in 2018 so that authors could publish their research findings as early as possible.
- Impact factor reached 2.57.
- Migrated to new and more author‐friendly submission system—ReX.
- Implemented image checking during peer review to ensure scientific integrity.

We believe that moving to Open Access will help us perform even better for our authors and our readers.

